# Serum sialic acid binding immunoglobulin-like lectin-1 (sSIGLEC-1) in Egyptian patients with lupus nephritis: correlation with renal activity in non-European ancestry

**DOI:** 10.1038/s41598-026-60610-x

**Published:** 2026-07-06

**Authors:** Samar Magdy, Mary Wadie, Alyaa Farid, Mohamed Nasser

**Affiliations:** 1 Immunology Section, Zoology Department, Faculty of Science, Cairo University, Giza, Egypt; 2 Rheumatology and Immunology Section, Faculty of Medicine, Cairo University, Giza, Egypt; 3 Biotechnology Department, Faculty of Science, Cairo University, Giza, Egypt

**Keywords:** Systemic lupus erythematosus, Lupus nephritis, sSIGLEC-1, Egyptian cohort, Non-European ancestry, Renal biopsy, CKD, Biomarkers, Diseases, Immunology, Nephrology, Rheumatology

## Abstract

Serum sialic acid binding immunoglobulin-like lectin-1 (sSIGLEC-1) is a type I interferon-associated biomarker previously linked to lupus nephritis (LN) in European ancestry populations, but its utility in non-European cohorts remains poorly defined. This study aimed to validate the association of sSIGLEC-1 with LN in Egyptian SLE patients (non-European ancestry) and to test its correlation with world health organization (WHO) pathological classes, chronic kidney disease (CKD) stages, systemic lupus international collaborating clinics-renal activity score (SLICC-RAS), systemic lupus erythematosus disease activity index (SLEDAI), 24-hour urinary protein and proinflammatory cytokines. This cross-sectional study included 80 SLE patients (47 with LN, 33 without LN) and 20 healthy controls. Renal biopsy was classified according to WHO criteria. Estimated glomerular filtration rate (eGFR) and CKD stages were calculated. Median levels of sSIGLEC-1 were significantly higher in SLE patients than controls (113.5 vs. 11.2 pg/mL) and in LN patients than non-LN patients (117.7 vs. 110.0 pg/mL). Multivariable logistic regression confirmed sSIGLEC-1 as an independent predictor of LN (OR = 1.02, *p* = 0.04). sSIGLEC-1 correlated positively with SLEDAI (*r* = 0.26), SLICC-RAS (*r* = 0.31), and proinflammatory cytokines (IL-1β, IL-6, TNF-α), but did not correlate with 24-hour urinary protein (*r* = − 0.175). However, no significant differences in sSIGLEC-1 were observed across WHO pathological classes or CKD stages. ROC analysis showed poor discriminative ability for LN (AUC = 0.6928, sensitivity 93.6%, specificity 39.4%). In Egyptian SLE patients, sSIGLEC-1 is elevated in LN and correlates with disease activity but does not reflect histological severity or chronic kidney damage. Its low specificity limits diagnostic utility; however, high sensitivity suggests potential as a rule-out screening test for LN in non-European populations.

## Introduction

Systemic lupus erythematosus (SLE) is a heterogeneous autoimmune disease characterized by loss of self-tolerance, leading to activation of B and T lymphocytes and production of various autoantibodies and cytokines. These immunological abnormalities result in immune complex deposition, ultimately causing tissue injury and inflammation in multiple organs. SLE manifests with variable complications depending on disease activity, including nephritis, central nervous system involvement, and anemia in severe forms of the disease^1,2^.

Lupus nephritis (LN) represents one of the most severe and frequent complications of SLE, frequently progressing to end-stage renal disease if inadequately treated. Initial diagnosis of LN relies on laboratory assessments including serum creatinine (Cr), urinary protein excretion (measured as 24-hour urinary protein or urine protein-to-creatinine ratio), and estimated glomerular filtration rate (eGFR), which provide valuable insights into kidney function and the extent of renal damage. However, although these markers are useful for monitoring disease progression and treatment response, they lack sufficient specificity to fully capture the underlying inflammatory processes driving LN. For definitive diagnosis and accurate evaluation of disease activity—including inflammation, fibrosis, and histopathological classification according to the World Health Organization (WHO) and the International Society of Nephrology/Renal Pathology Society (ISN/RPS) classification—renal biopsy remains the gold standard. This invasive procedure allows direct examination of renal tissue, enabling clinicians to determine disease severity, guide targeted therapy, and predict long-term outcomes^3^. Thus, despite the utility of non-invasive biomarkers, renal biopsy remains indispensable for comprehensive LN management.

Sialic acid binding immunoglobulin-like lectin-1 (SIGLEC-1) belongs to the family of animal cell surface glycan-binding proteins known as SIGLECs, of which humans express fourteen distinct types. SIGLECs are divided into two classes: the first class comprises thirteen types found on various immune cells functioning as immune regulatory molecules, while the second class consists of a single type (SIGLEC-4) expressed on myelinating neurons^4–6^. SIGLEC-1 (also known as CD169) is primarily expressed on the surface of macrophages and plays crucial roles in binding sialylated antigens, antigen presentation, and regulating immune responses against self-antigens^7,8^. Additionally, SIGLEC-1 exists as a soluble form (sSIGLEC-1) in serum/plasma, restricted to CD14⁺ monocytes, and its expression is elevated in various autoimmune diseases^9–12^.

A previous study confirmed a positive association between both soluble and membrane-bound forms of SIGLEC-1 in SLE patients. The same study also found that sSIGLEC-1 levels increase in the presence of renal complications among SLE patients of European ancestry, suggesting a relationship between sSIGLEC-1 levels and ancestry differences^13^. However, this hypothesis remained tentative due to the limited number of non-European subjects included in that analysis. Another study reported elevated sSIGLEC-1 levels in Egyptian SLE patients, but it did not examine correlations with WHO pathological classes, chronic kidney disease (CKD) stages, or detailed renal activity scores^14^.

The present study aimed to validate the association of sSIGLEC-1 with LN in an Egyptian cohort (representing non-European ancestry) and to comprehensively test whether sSIGLEC-1 correlates with clinically relevant parameters that have not been previously examined in non-European populations. These parameters include WHO pathological classes, CKD stages, the systemic lupus international collaborating clinics renal activity score (SLICC-RAS), systemic lupus erythematosus disease activity index (SLEDAI), 24-hour urinary protein and proinflammatory cytokines. Unlike previous investigations, this study explicitly reports both positive and negative findings to accurately define the ancestry-specific clinical utility of sSIGLEC-1. To our knowledge, this is the first dedicated validation of sSIGLEC-1 as a potential associative marker for LN in a well-characterized non-European (Egyptian) cohort, incorporating detailed histopathological classification and renal function staging. Importantly, while our sample size is modest, a post-hoc power calculation confirms adequate power (80%) for the primary comparison between LN and non-LN patients, and all negative findings are explicitly reported as exploratory, requiring future validation in larger cohorts.

## Patients and methods

### Study design and participants

A cross-sectional study was conducted from January 2021 to February 2022, enrolling 80 SLE patients (68 females, 12 males) with a mean age of 36.3 ± 8.64 years and mean disease duration of 34.8 ± 20.92 months. Patients were recruited from the Outpatient Clinic of Immunology and Rheumatology at Kaser Al-Ainy Hospital, Cairo University, following the American College of Rheumatology/European League Against Rheumatism (ACR/EULAR) classification criteria^15^. Based on renal involvement, patients were divided into two groups: 47 SLE patients with LN and 33 patients without LN. The definition and diagnosis of LN were established by the presence of proteinuria > 0.5 g/day combined with histological examination of renal biopsy^16^. Exclusion criteria included conditions that could confound LN assessment or influence renal pathology: (1) diabetes mellitus (risk of diabetic nephropathy), (2) chronic infections (potential for immune-mediated renal injury or disease mimicry), (3) hypertension (to exclude hypertensive nephrosclerosis), and (4) overlap autoimmune disorders (to maintain a homogeneous study population). Additionally, 20 age- and sex-matched healthy individuals were enrolled as controls.

The study adhered to the Strengthening the Reporting of Observational Studies in Epidemiology (STROBE) guidelines^17^ and complied with the principles of the Declaration of Helsinki. Written informed consent was obtained from all participants, and the study protocol was approved by the Research Ethics Committee of the Faculty of Medicine, Cairo University, Egypt (approval number: S-14-2019).

### Disease activity evaluation

Following comprehensive clinical examination, all SLE patients underwent detailed medical history assessment. Disease activity was quantitatively evaluated using SLEDAI, a validated scoring system with a maximum possible score of 105 points. According to established clinical criteria, patients scoring ≥ 12 points were classified as having active disease^18^. For specific assessment of renal involvement, SLICC-RAS was employed. This scoring system assigns weighted points based on urinary findings: 1 point for white blood cells > 5/high-power field (hpf), 3 points for red blood cells > 5/hpf or proteinuria (0.5–1 g/day), 5 points for proteinuria (1–3 g/day), and 11 points for proteinuria > 3 g/day^19^. This granular scoring system enabled precise stratification of renal disease severity in our patient cohort.

### Laboratory assessment

Approximately 5 mL of venous blood was collected from all subjects between 8:00–10:00 AM to minimize circadian variation. Serum was separated by centrifugation at 1500 × g for 10 min at 4 °C within 2 h of collection, aliquoted into cryovials, and stored at − 80 °C until analysis. All samples were subjected to a maximum of two freeze-thaw cycles. Urine samples were collected on the same day as blood collection for urinalysis and 24-hour urinary protein estimation. Laboratory personnel performing enzyme-linked immunosorbent assay (ELISA) measurements were blinded to patient clinical status (SLE with LN, SLE without LN, or healthy control). Clinical data (SLEDAI, SLICC-RAS, eGFR, 24-hour urinary protein) were collected and recorded by a separate investigator who was unaware of sSIGLEC-1 results. Renal biopsy histopathological classification (WHO class) was performed by an experienced pathologist who was blinded to both clinical and laboratory data. Unblinding occurred only after all measurements and classifications were completed. The following commercially available ELISA kits were used according to the manufacturers’ instructions:

**Table:** 

Analyte	Kit manufacturer	Catalog number	Detection limit	Intra-assay CV (%)	Inter-assay CV (%)
ANA	Mybiosource (Canada)	MBS702970	0.1 ng/ml	< 15	< 15
Anti-dsDNA	Mybiosource (Canada)	MBS269122	Up to 1 IU/mL	≤ 8	≤ 12
C3	Elabscience (USA)	E-EL-H6054	0.94 ng/mL	< 10	< 10
C4	Elabscience (USA)	E-EL-H6309	0.19 ng/mL	< 10	< 10
Serum Cr	Elabscience (USA)	E-EL-0058	0.75 µg/mL	< 10	< 10
sSIGLEC-1	Assaygenie (London)	HUF100069	0.094 ng/mL	< 8	< 10
IL-1β	ElK Biotechnology (USA)	ELK1270	5.8 pg/mL	< 8	< 10
IL-6	ElK Biotechnology (USA)	ELk1156	3.3 pg/mL	< 8	< 10
TNF-α	ElK Biotechnology (USA)	ELK1190	6.4 pg/mL	< 8	< 10

CV, coefficient of variation; ANA, anti-nuclear antibody; Anti-dsDNA, anti-double-stranded deoxyribonucleic acid; C3, complement 3; C4, complement 4; Cr, creatinine; sSIGLEC-1, serum sialic acid binding immunoglobulin-like lectin-1; IL-1β, interleukin-1beta; IL-6, interleukin-6; TNF-α, tumor necrosis factor-alpha.

All ELISA plates were read using a microplate reader at 450 nm with wavelength correction at 570 nm. Each sample was measured in duplicate, and the mean value was used for analysis. Samples with a duplicate coefficient of variation (CV) > 10% were re-analyzed.

### eGFR calculations

eGFR was calculated using the following formula^20,21^:

$${\text{eGFR }}({\mathrm{ml}}/{\mathrm{min}}/{\mathrm{1}}.{\text{73 m}}^{{\mathrm{2}}} ) = {\text{194 X }}\left( {{\text{Cr }}\left( {{\mathrm{mg}}/{\mathrm{dl}}} \right)} \right)^{{ - {\mathrm{1}}.0{\mathrm{94}}}} {\text{X }}\left( {{\mathrm{age}}} \right)^{{ - 0.{\mathrm{287}}}} \left( {{\text{for males}}} \right)$$

$${\text{eGFR }}({\mathrm{ml}}/{\mathrm{min}}/{\mathrm{1}}.{\text{73 m}}^{{\mathrm{2}}} ) = {\text{194 X }}\left( {{\text{Cr }}\left( {{\mathrm{mg}}/{\mathrm{dl}}} \right)} \right)^{{ - {\mathrm{1}}.0{\mathrm{94}}}} {\text{X }}\left( {{\mathrm{age}}} \right)^{{ - 0.{\mathrm{287}}}} {\text{X }} 0.{\text{739 }}\left( {{\text{for females}}} \right)$$

### Classification according to CKD stages

According to the chronic kidney disease (CKD) criteria based on the Kidney Disease Outcomes Quality Initiative (K/DOQI) guidelines of the National Kidney Foundation^22^, SLE patients were classified into five CKD stages as follows:

- *Stage I:* eGFR > 90 mL/min/1.73 m².
- *Stage II: *90 mL/min/1.73 m² < eGFR ≥ 60 mL/min/1.73 m².
- *Stage III: *59 mL/min/1.73 m² < eGFR ≥ 30 mL/min/1.73 m².
- *Stage IV:* 29 mL/min/1.73 m² < eGFR ≥ 15 mL/min/1.73 m².
- *Stage V:* eGFR < 15 mL/min/1.73 m².

### Renal biopsy

Renal biopsy samples were obtained from LN patients and analyzed by an experienced pathologist who was blinded to the patients’ clinical conditions. Histological examination was performed using light microscopy and immunofluorescence, and biopsies were categorized according to the WHO classification of LN^23^.

### Statistical analysis

Normality of continuous variables was assessed using the Kolmogorov–Smirnov test. For comparisons between two groups, the independent sample t-test (for normally distributed variables) or the Mann–Whitney U test (for non-normally distributed variables) was used. For comparisons involving more than two groups, the Kruskal–Wallis test with post-hoc Dunn’s test (for non-parametric data) or one-way ANOVA with post-hoc Tukey’s test (for parametric data) was applied. Correlations were assessed using Spearman’s rank correlation test. To determine whether sSIGLEC-1 is independently associated with LN after adjusting for potential confounders, multivariable logistic regression was performed with LN status (present vs. absent) as the dependent variable. Independent variables included sSIGLEC-1 level, age, sex, disease duration, and treatment (prednisone, entered as binary variables). Receiver operating characteristic (ROC) curve analysis was performed to evaluate the ability of sSIGLEC-1 to discriminate between SLE patients with and without LN. The 95% confidence intervals for the area under the curve (AUC) were calculated using the Hanley and McNeil approach^24^, and the optimal cut-off value was determined by maximizing the Youden index (sensitivity + specificity − 1)^25^. No adjustment for multiple comparisons was applied to the Kruskal–Wallis tests (WHO classes and CKD stages), as these were exploratory analyses. A *p* value ≤ 0.05 was considered statistically significant.

## Results

### Baseline demographic and clinical characteristics of SLE patients

The baseline demographic and clinical characteristics of the 80 SLE patients are summarized in Table 1. SLEDAI score was 23.1 ± 6.25, and the mean of SLICC-RAS was 5.05 ± 3.31. The mean erythrocyte sedimentation rate (ESR) at the first hour was 36.3 ± 18.11 mm/hr. Anemia, malar rash, and neurological symptoms were observed in 73 (91.25%), 15 (18.75%), and 32 (40%) of patients, respectively. All SLE patients (100%) tested positive for ANA and anti-dsDNA. Low complement levels (C3 < 80 mg/dl and C4 < 12 mg/dl) were observed in 27 (33.75%) and 24 (30%) patients, respectively.

**Table 1:** Demographic and clinical properties of the studied SLE patients.

Feature	Value
Sex (Women/Men)	68/12
Age (years; mean ± SD)	36.3 ± 8.64
Disease duration (months; mean ± SD)	34.8 ± 20.92
SLEDAI (mean ± SD)	23.1 ± 6.25
SLICC-RAS (mean ± SD)	5.05 ± 3.31
ESR (mm/hr) (mean ± SD)	36.3 ± 18.11
Malar rash (n/%)	15/18.75
Neurologic complications (n/%)	32/40
Anemia (n/%)	73/91.25
ANA positive (n/%)	80/100
Anti-dsDNA positive (n/%)	80/100
C3 < 80 mg/dl (n/%)	27/33.75
C4 < 12 mg/dl (n/%)	24/30

SLEDAI, systemic lupus erythematosus disease activity index; SLICC-RAS, systemic lupus international collaborating clinics-renal activity score; ESR, erythrocyte sedimentation rate; ANA, anti-nuclear antibody; Anti-dsDNA, anti-double-stranded deoxyribonucleic acid; C3, complement 3; C4, complement 4. Data are presented as mean ± standard deviation (SD) or number/percentage (n/%).

### SLE patients and healthy controls

As shown in Table 2, no significant differences were observed between SLE patients (*n* = 80) and healthy controls (*n* = 20) regarding sex distribution or age (*p* > 0.05 for both). However, SLE patients exhibited significantly higher values in several clinical and laboratory parameters. Specifically, serum Cr levels were markedly elevated in SLE patients (2.69 ± 1.99 mg/dl) compared to controls (0.72 ± 0.18 mg/dl). The median 24-hour urinary protein excretion was significantly higher in SLE patients [3.3 (0.48–5.4) g/day] than in controls [0.3 (0.22–0.34) g/day]. Conversely, eGFR was significantly lower in SLE patients [22.29 (11.68–60.47) ml/min/1.73 m²] compared to controls [81.70 (63.48–107.79) ml/min/1.73 m²]. Furthermore, serum sSIGLEC-1 levels were significantly elevated in SLE patients [113.5 (96.8–120.0) pg/mL] relative to healthy controls [11.15 (10.22–13.27) pg/mL].

**Table 2:** Demographic and clinical differences between SLE patients and control groups.

Parameters	SLE Patients (*n* = 80)	Control (*n* = 20)
Sex (M/F)	12/68	6/14
Age (years)	36.3 ± 8.64	38.0 ± 5.98
Serum Cr (mg/dl)	2.69 ± 1.99^a^	0.72 ± 0.18
24-hour urinary protein (g/day)	3.3 (0.48–5.4)^a^	0.3 (0.22–0.34)
eGFR (ml/min/1.73 m^2^)	22.29 (11.68–60.47)^a^	81.70 (63.48-107.79)
sSIGLEC-1 (pg/mL)	113.5 (96.8–120.0)^a^	11.15 (10.22–13.27)

Cr, creatinine; eGFR, estimated glomerular filtration rate; sSIGLEC-1, serum sialic acid binding immunoglobulin-like lectin-1.^a^Denotes significant difference between SLE patients and control group. Data are presented as mean ± standard deviation or median (interquartile range). A *p* value ≤ 0.05 was considered statistically significant.

### SLE patients with and without LN

No significant differences were found between SLE patients with LN (*n* = 47) and those without LN (*n* = 33) regarding sex, age, or disease duration (*p* > 0.05 for all), as detailed in Table 3. However, patients with LN exhibited significantly higher values for several parameters compared to those without LN: SLEDAI (27.78 ± 1.81 vs. 16.42 ± 3.69), SLICC-RAS [5.0 (5–11) vs. 3.0 (1–3)], serum Cr (3.97 ± 1.64 vs. 0.87 ± 0.38 mg/dl), and 24-hour urinary protein (5.30 ± 1.74 vs. 0.44 ± 0.06 g/day). In contrast, patients without LN had a significantly higher eGFR (76.94 ± 34.38 ml/min/1.73 m²) compared to those with LN (14.75 ± 7.51 ml/min/1.73 m²). Additionally, serum sSIGLEC-1 levels were significantly higher in LN patients [117.7 (109.3–144.2) pg/mL] than in non-LN patients [110.0 (90.525–118.05) pg/mL].

**Table 3:** Demographic and clinical differences between SLE patients with and without LN.

Parameters	Patients with LN (*n* = 47)	Patients without LN (*n* = 33)
Sex (M/F)	5/42	7/26
Age (years)	37.44 ± 9.03	34.66 ± 7.88
Disease duration (months)	24.0 (20–48)	36.0 (17-47.5)
SLEDAI	27.78 ± 1.81^a^	16.42 ± 3.69
SLICC-RAS	5.0 (5–11)^a^	3.0 (1–3)
Serum Cr (mg/dl)	3.97 ± 1.64^a^	0.87 ± 0.38
24-hour urinary protein (g/day)	5.30 ± 1.74^a^	0.44 ± 0.06
eGFR (mL/min/1.73 m^2^)	14.75 ± 7.51^a^	76.94 ± 34.38
sSIGLEC-1 (pg/mL)	117.7 (109.3-144.2)^a^	110.0 (90.525–118.05)

LN, lupus nephritis; SLEDAI, systemic lupus erythematosus disease activity index; SLICC-RAS, systemic lupus international collaborating clinics-renal activity score; Cr, creatinine; eGFR, estimated glomerular filtration rate; sSIGLEC-1, serum sialic acid binding immunoglobulin-like lectin-1.^a^Denotes significant difference between SLE patients with and without LN. Data are presented as mean ± standard deviation or median (interquartile range). A *p* value ≤ 0.05 was considered statistically significant.

### Multivariable logistic regression analysis for factors associated with LN

A multivariable logistic regression analysis was performed to identify factors independently associated with LN in Egyptian SLE patients (Table 4). The model included sSIGLEC-1 level, age, sex, disease duration, and treatment (prednisone) as independent variables, with LN status (present vs. absent) as the dependent variable. After adjustment for covariates, sSIGLEC-1 level showed a statistically significant positive association with LN (coefficient = 0.02063, OR = 1.0208, 95% CI 1.0009–1.0412, *p* = 0.0405). This indicates that each 1 pg/mL increase in sSIGLEC-1 level was associated with approximately 2.1% higher odds of having LN. Treatment (prednisone) was strongly and significantly associated with LN (coefficient = 2.314, OR = 10.11, 95% CI 1.936–52.8, *p* = 0.0061), indicating that patients receiving immunosuppressive therapy had approximately 10-fold higher odds of having LN, which is expected given that LN requires active treatment. Age (*p* = 0.1669), sex (*p* = 0.938), and disease duration (*p* = 0.7447) were not statistically significant predictors of LN in this model.

**Table 4:** Multivariable logistic regression analysis for factors associated with LN in SLE patients.

Variables	Coefficient	OR	95% CI	*P* value
sSIGLEC-1 (pg/mL)	0.02063	1.0208	(1.0009–1.0412)	0.0405
Age (years)	0.04475	1.0458	(0.9815–1.114)	0.1669
Sex	− 0.05974	0.942	(0.209–4.243)	0.938
Disease duration (months)	0.0039	1.004	(0.9804–1.028)	0.7447
treatment	2.314	10.11	(1.936–52.8)	0.006083

OR, odd ratio; CI, confidence interval; sSIGLEC-1, serum sialic acid binding immunoglobulin-like lectin-1. Dependent variable: LN status (0 = no LN, 1 = LN). A *p* value ≤ 0.05 was considered statistically significant.

### ROC curve analysis of sSIGLEC-1 for discriminating LN

**Fig. 1:**
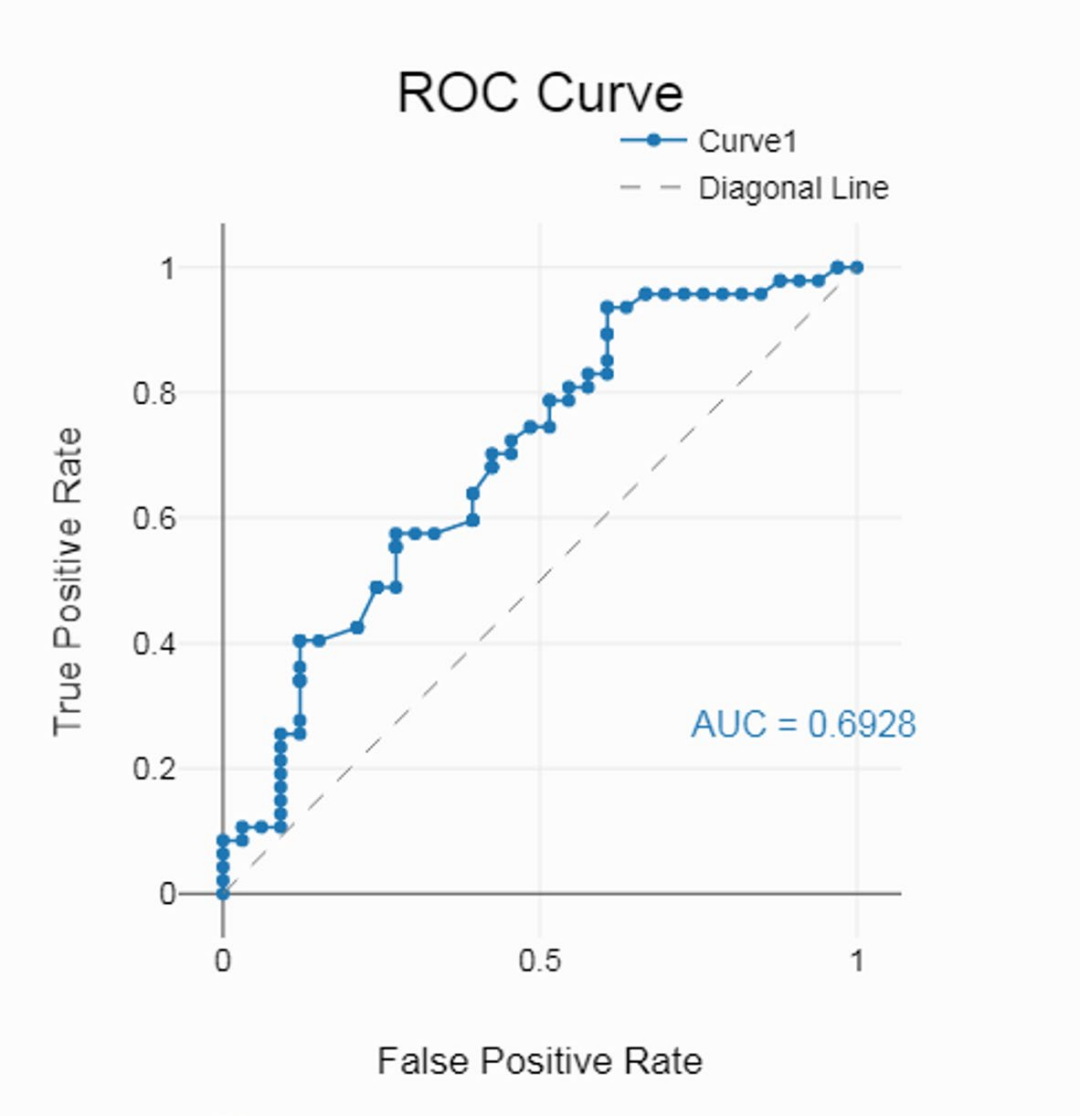
Receiver operating characteristic (ROC) curve showing the performance of serum sSIGLEC-1 levels in distinguishing SLE patients with LN (*n* = 47) from those without LN (*n* = 33). The area under the curve (AUC) was 0.6928 (95% CI 0.52–0.87, *P =* 0.03).

Receiver operating characteristic (ROC) curve analysis was performed to evaluate the ability of serum sSIGLEC-1 levels to discriminate between SLE patients with and without LN (Fig. 1). The area under the curve (AUC) was 0.6928 (95% CI 0.52–0.87, *p* = 0.03), indicating poor to fair discriminative ability. Using the optimal cut-off value determined from the ROC curve coordinates (Youden index), the diagnostic performance of sSIGLEC-1 was as follows: Optimal cut-off: 110.3 pg/mL, Sensitivity: 93.6% (44 out of 47 LN patients correctly identified), Specificity: 39.4% (13 out of 33 non-LN patients correctly identified), Positive predictive value (PPV): 68.8%, Negative predictive value (NPV): 81.3%, and Accuracy: 71.3%. At this cut-off, the false positive rate was 60.6%, and the true positive rate (sensitivity) was 93.6%.

### sSIGLEC-1 levels across WHO pathological classes of LN

Patients diagnosed with LN (*n* = 47) were classified into five WHO classes based on histological examination of renal biopsy samples. The distribution was as follows: class I, 11 patients (23.4%); class II, 5 patients (10.63%); class III, 3 patients (6.4%); class IV, 24 patients (51.06%); and class V, 4 patients (8.51%). Class IV was the most frequent. As shown in Table 5, the median sSIGLEC-1 levels did not differ significantly among the five WHO classes. These results indicate that sSIGLEC-1 levels are not associated with histopathological severity in LN.

**Table 5:** Difference in the level of sSIGLEC-1 between WHO pathological classes of LN.

Parameter	Class I (*n* = 11)	Class II (*n* = 5)	Class III (*n* = 3)	Class IV (*n* = 24)	Class V (*n* = 4)
sSIGLEC-1 (pg/mL)	117.7 (112.9–152.0)	118.1 (91.55–154.2)	112.0 (95.60–118.10)	115.5 (105.37–138.23)	140.85 (111.25–199.85)

sSIGLEC-1, serum sialic acid binding immunoglobulin-like lectin-1. Data are presented as median (interquartile range). A *p* value ≤ 0.05 was considered statistically significant.

### sSIGLEC-1 levels across CKD stages

All SLE patients (*n* = 80) were systematically categorized into five stages of CKD according to the 2002 K/DOQI guidelines. The distribution was as follows: stage I, 11 patients; stage II, 10 patients; stage III, 12 patients; stage IV, 19 patients; stage V, 28 patients. As expected, eGFR values differed significantly between stages (Table 6). However, analysis of sSIGLEC-1 levels revealed no significant differences among the five CKD stages, indicating that sSIGLEC-1 does not reflect the degree of chronic renal impairment.

**Table 6:** Difference in the level of sSIGLEC-1 between different CKD stages.

Parameter	Stage I (*n* = 11)	Stage II (*n* = 10)	Stage III (*n* = 12)	Stage IV (*n* = 19)	Stage V (*n* = 28)
eGFR (mL/min/1.73 m^2^)	118.40 ± 18.03	71.87 ± 7.26^a^	44.12 ± 9.35^ab^	21.56 ± 4.73^abc^	9.71 ± 2.76^abcd^
sSIGLEC-1 (pg/mL)	112.4(85.4-118.20)	112.3(105.72-122.07)	106.85(91.14-117.92)	118.10(101.11–120.3)	115.5(106.52-150.12)

sSIGLEC-1, serum sialic acid binding immunoglobulin-like lectin-1; eGFR, estimated glomerular filtration rate. Data are presented as mean ± standard deviation (for eGFR) or median (interquartile range) (for sSIGLEC-1). Letters a, b, c, and d denote significant difference compared to stage I, stage II, stage III, and stage IV, respectively (*p* ≤ 0.05).

### Correlation analysis of sSIGLEC-1 with disease activity indices and proinflammatory cytokines

Spearman correlation analysis was performed to evaluate the relationships between serum sSIGLEC-1 levels and disease activity indices, 24-hour urinary protein, and proinflammatory cytokines in SLE patients (Table 7). sSIGLEC-1 showed a significant positive correlation with SLEDAI (*r* = 0.263) and with SLICC-RAS (*r* = 0.305). No significant correlation was observed between sSIGLEC-1 and 24-hour urinary protein (*r* = -0.175). Additionally, sSIGLEC-1 demonstrated significant positive correlations with all three proinflammatory cytokines measured: IL-1β (*r* = 0.245), IL-6 (*r* = 0.324), and TNF-α (*r* = 0.322). These findings indicate that higher serum sSIGLEC-1 levels are associated with increased disease activity and elevated proinflammatory cytokine levels in Egyptian SLE patients. These findings suggest that sSIGLEC-1 reflects systemic type I interferon activity rather than glomerular protein leakage.

**Table 7:** Correlations of sSIGLEC-1 with disease activity indices, 24-hour urinary protein and proinflammatory cytokines.

Parameters	sSIGLEC-1 (pg/mL)
	Spearman (*r*)
SLEDAI	0.263*
SLICC-RAS	0.305*
24-hour urinary protein (g/day)	− 0.175
IL-1β (pg/mL)	0.245*
IL-6 (pg/mL)	0.324*
TNF-α (pg/mL)	0.322*

sSIGLEC-1, serum sialic acid binding immunoglobulin-like lectin-1; SLEDAI, systemic lupus erythematosus disease activity index; SLICC-RAS, systemic lupus international collaborating clinics-renal activity score; IL-1β, interleukin-1beta; IL-6, interleukin-6; TNF-α, tumor necrosis factor-alpha. Data are presented as Spearman correlation coefficient (r). An asterisk (*) denotes a significant correlation (*p* ≤ 0.05).

## Discussion

SLE is known for its heterogeneous clinical manifestations, which arise from the activation of various lymphokine systems during the disease course due to abnormal production of autoantibodies and cytokines by activated B and T helper (Th) lymphocytes^26^. CKD is one of the most common complications of SLE and is recognized as a risk factor for cardiovascular disease and end-stage renal disease in these patients. LN is a frequent cause of mortality in SLE, with approximately 50% of patients developing LN during the disease course^27^. The pathogenesis of LN is associated with abnormal production of type I interferon (IFN-I), a condition known as interferonopathy^28,29^. The primary objectives of this study were twofold: first, to validate the association between sSIGLEC-1 and LN in an Egyptian (non-European) cohort, and second, to assess whether sSIGLEC-1 correlates with previously unexplored clinical parameters in non-European populations, including WHO classes, CKD stages, SLICC-RAS, SLEDAI, 24-hour urinary protein and proinflammatory cytokines. This represents the first dedicated validation of sSIGLEC-1 as a potential LN biomarker in a well-characterized non-European (Egyptian) cohort incorporating both histopathological and renal functional assessments. Unlike prior studies, we explicitly report both positive and negative results to accurately define the ancestry-specific clinical utility of sSIGLEC-1.

Identifying biomarkers associated with LN is important for understanding disease mechanisms and guiding clinical research. The landmark study by Oliveira et al.^13^ first identified sSIGLEC-1 as a plasma biomarker associated with the type I interferon signature and renal disease in SLE. In a large cohort of 656 SLE patients, they reported that higher sSIGLEC-1 concentrations were associated with an increased frequency of renal complications, particularly in patients of European ancestry. However, Oliveira et al. noted that non-European patients had higher baseline sSIGLEC-1 levels than European patients, but the association with renal disease was less pronounced in their non-European subgroup due to limited sample size. They explicitly stated that their non-European sample was too small for definitive conclusions. Our study addresses this gap by providing the first dedicated validation in a large Egyptian cohort. We confirm that sSIGLEC-1 is elevated in SLE patients and is significantly higher in those with LN. Furthermore, our multivariable analysis extends Oliveira’s findings by demonstrating that sSIGLEC-1 independently predicts LN in a non-European population with a similar effect size (OR ≈ 1.02 per pg/mL).

Our analysis of demographic data showed no significant difference in sex distribution between LN and non-LN patients, which agrees with Flower et al.^30^ but contrasts with Al Attia^31^, who reported that nephritis is more common in male SLE patients than females. No significant difference in age between LN and non-LN patients was found in our study, consistent with Mohsen et al.^32^ but contrasting with Kosalka-Wegiel et al.^33^. Similarly, we observed no significant difference in disease duration between the two patient groups, consistent with both Mohsen et al.^32^ and Kosalka-Wegiel et al.^33^. These demographic findings suggest that LN risk in Egyptian SLE patients is not strongly associated with age, sex, or disease duration, which aligns with the results of our multivariable regression model.

Our multivariable analysis showed that treatment (prednisone) was strongly associated with LN. This is an expected finding, as patients with active LN require immunosuppressive therapy, while SLE patients without renal involvement may be managed with milder regimens (e.g., hydroxychloroquine alone)^34^. This finding also serves as a positive control, validating that our model correctly identifies known clinical associations. However, it also highlights a potential confounding effect: treatment may influence sSIGLEC-1 levels independently of disease activity. Biesen et al.^35^ previously reported that SIGLEC-1 expression decreases with successful therapy. Our cross-sectional design cannot determine whether elevated sSIGLEC-1 in LN patients reflects disease activity, treatment resistance, or a combination of both. Prospective studies measuring sSIGLEC-1 before and after treatment initiation are needed to address this question.

The present study demonstrated significant positive correlations between sSIGLEC-1 and both SLEDAI (*r* = 0.263) and SLICC-RAS (*r* = 0.305). These findings are particularly noteworthy because Oliveira et al.^13^ previously reported that sSIGLEC-1 levels were not associated with the SLEDAI clinical score in their multi-ethnic cohort. This discrepancy may be explained by ancestry-related differences. Oliveira et al. found that non-European SLE patients had higher baseline sSIGLEC-1 levels compared to European patients and suggested that disease heterogeneity in non-European populations could reflect a reduced dependency on the type I IFN pathway for disease severity. Our findings in an Egyptian population suggest that, contrary to Oliveira’s observations in European cohorts, sSIGLEC-1 does correlate with global disease activity in this non-European population. Recent work by Böni et al.^36^ demonstrated that CD169 (SIGLEC-1) expression on peripheral blood monocytes correlates with biopsy-proven renal manifestation in SLE patients. They also reported that CD169 expression positively correlated with disease activity (SELENA-SLEDAI). These findings support the biological plausibility of sSIGLEC-1 as a marker of renal involvement, as CD169 is the membrane-bound form of SIGLEC-1 from which the soluble form (sSIGLEC-1) is derived. The correlation with SLICC-RAS, a specific measure of renal activity, is consistent with the known role of SIGLEC-1 in renal inflammation. SIGLEC-1 is expressed on macrophages infiltrating inflamed tissues, including the kidney, and participates in antigen presentation and immune response regulation^37^. Our finding that sSIGLEC-1 levels reflect both global disease activity (SLEDAI) and specific renal activity (SLICC-RAS) in Egyptian SLE patients supports the assertion that this biomarker is integrated into the inflammatory network driving LN.

The significant positive correlations between sSIGLEC-1 and the proinflammatory cytokines IL-1β (*r* = 0.245), IL-6 (*r* = 0.324), and TNF-α (*r* = 0.322) provide important mechanistic insights. sSIGLEC-1 is a type I interferon-regulated biomarker expressed on monocytes and macrophages^13^. Type I interferon signaling is known to potentiate the production of proinflammatory cytokines including IL-6 and TNF-α in SLE. The positive correlations between sSIGLEC-1 and these cytokines suggest that the type I IFN signature (reflected by sSIGLEC-1) is associated with a broader inflammatory state characterized by elevated IL-1β, IL-6, and TNF-α. These findings are consistent with the known role of SIGLEC-1 in inflammatory responses through cell-cell interactions^37^ and support the biological plausibility of sSIGLEC-1 as a marker of active inflammation in LN.

An important negative finding in this study was the absence of a significant correlation between sSIGLEC-1 and 24-hour urinary protein excretion (*r* = -0.175). While 24-hour urinary protein is a direct measure of glomerular damage and a key criterion for diagnosing and monitoring LN^16^, our results indicate that sSIGLEC-1 does not reflect the magnitude of proteinuria. This dissociation is biologically plausible for several reasons. First, sSIGLEC-1 is a type I interferon-inducible marker expressed on monocytes and macrophages, reflecting systemic immune activation rather than local glomerular injury^13^. In contrast, proteinuria results from structural damage to the glomerular filtration barrier, including podocyte injury, loss of charge selectivity, and disruption of the slit diaphragm. A patient may have severe podocyte injury with massive proteinuria but only moderate monocyte activation, or conversely, strong type I IFN signaling with minimal proteinuria if the glomerular basement membrane remains intact. Second, previous studies have shown that proteinuria correlates better with chronic histopathological changes (e.g., glomerulosclerosis, fibrous crescents) than with acute inflammatory activity^23^. Since sSIGLEC-1 reflects acute type I IFN-driven inflammation rather than chronic damage, the lack of correlation with proteinuria is consistent with our other negative findings (no association with WHO classes or CKD stages). Third, this finding has clinical implications. A patient with suspected LN who presents with elevated sSIGLEC-1 but low proteinuria may have early or mild glomerular involvement, or predominantly extra-renal disease activity. Conversely, a patient with high proteinuria but normal sSIGLEC-1 may have chronic, smoldering LN or non-inflammatory proteinuria from other causes. Therefore, sSIGLEC-1 and 24-hour urinary protein provide complementary, non-redundant information. Neither should replace the other; rather, they should be interpreted together along with other clinical and laboratory parameters. Our results are consistent with Oliveira et al.^13^, who also found that sSIGLEC-1 was associated with renal disease but did not report a strong correlation with proteinuria in their non-European subgroup. The weak negative direction of the correlation (*r* = -0.175) in our study suggests a possible trend toward lower sSIGLEC-1 in patients with higher proteinuria, which could reflect treatment effects (patients with heavy proteinuria are more aggressively immunosuppressed, potentially lowering sSIGLEC-1) or disease chronicity (burned-out LN with persistent proteinuria but quiescent inflammation). However, given the non-significant *p* value, this should be interpreted with caution and explored in future prospective studies.

A critical and novel contribution of our study is the demonstration that sSIGLEC-1 levels do not differ across WHO pathological classes of LN or across CKD stages. These negative findings are important because they define the ancestry-specific utility of sSIGLEC-1. Previous studies, including Oliveira et al.^13^, did not examine these correlations in non-European populations. Our results indicate that while sSIGLEC-1 is elevated in LN and correlates with disease activity, it does not reflect the histopathological severity of renal injury (proliferative vs. membranous classes) nor the degree of chronic kidney damage (CKD stages I–V). This suggests that sSIGLEC-1 is a marker of active inflammation and type I IFN signaling rather than a marker of irreversible tissue damage or fibrosis. This distinction is clinically important: a marker that correlates with activity but not chronicity could be useful for detecting flares but not for staging long-term renal impairment. Our finding that class IV LN was the most frequent (51.06%) among Egyptian LN patients is consistent with Faezi et al.^38^, who also found class IV to be the most common in their cohort. However, the lack of association between sSIGLEC-1 and WHO classes indicates that this marker cannot replace renal biopsy for histopathological classification. Renal biopsy remains indispensable for guiding therapy, as proliferative classes (III and IV) require more aggressive immunosuppression than membranous class V.

ROC curve analysis demonstrated that sSIGLEC-1 has poor to fair discriminative ability for LN (AUC = 0.6928). An AUC of 0.6928 indicates that sSIGLEC-1 is only slightly better than random chance at distinguishing LN from non-LN. This finding is consistent with Oliveira et al.^13^, who reported that sSIGLEC-1 concentrations were associated with renal disease but did not demonstrate strong diagnostic accuracy. At the optimal cut-off of 110.3 pg/ml, sSIGLEC-1 showed high sensitivity (93.6%) but low specificity (39.4%). The high sensitivity suggests that sSIGLEC-1 could be useful as a rule-out screening test for LN: low levels (≤ 110.3 pg/ml) may confidently exclude LN, as evidenced by the negative predictive value of 81.3%. However, the low specificity indicates that elevated sSIGLEC-1 levels are not specific to LN and may be elevated in other conditions associated with type I interferon activation (e.g., viral infections, other autoimmune diseases), leading to a high false positive rate (60.6%). The positive predictive value (68.8%) indicates that approximately 69% of patients with elevated sSIGLEC-1 (> 110.3 pg/ml) actually have LN, while the remaining 31% would be false positives. Taken together, these findings indicate that sSIGLEC-1 should not be used as a standalone diagnostic test for LN but may have value as a rule-out screening tool to identify patients who require further evaluation (e.g., renal biopsy).

Our study was specifically designed to validate sSIGLEC-1 in a non-European (Egyptian) population. The results provide several important ancestry-specific insights. First, we confirmed that sSIGLEC-1 levels are elevated in Egyptian SLE patients compared to controls, consistent with Oliveira et al.^13^ who noted that non-European patients had higher baseline sSIGLEC-1 levels than European patients. Second, contrary to Oliveira’s observation that the association with renal disease was less pronounced in non-Europeans, we found a significant association between sSIGLEC-1 and LN in Egyptians, with a similar effect size to the European subgroup in Oliveira’s study. Third, we observed significant correlations with SLEDAI and SLICC-RAS that were not present in Oliveira’s European cohort, suggesting ancestry-specific differences in the relationship between sSIGLEC-1 and disease activity. These findings highlight the importance of conducting biomarker validation studies in diverse populations, as results from European cohorts may not directly generalize to non-European ancestries. Also, our study has several clinical implications. For clinicians managing Egyptian SLE patients, a low sSIGLEC-1 level (≤ 110.3 pg/ml) may help rule out LN with reasonable confidence (NPV 81.3%), potentially reducing the number of unnecessary renal biopsies in low-risk patients. However, an elevated sSIGLEC-1 level should not be used alone to diagnose LN or to guide therapy; given the high false positive rate (60.6%) and lack of association with WHO classes. sSIGLEC-1 is not a substitute for renal biopsy, which remains the gold standard for histopathological classification and treatment decisions. Additionally, the lack of association with CKD stages indicates that sSIGLEC-1 cannot be used to stage chronic kidney damage or predict long-term renal outcomes.

Based on our findings, several future research directions are warranted. First, prospective longitudinal studies should examine whether sSIGLEC-1 levels change with LN flares and treatment response. Second, multi-center studies across different non-European populations (African, Asian, Latin American) are needed to determine whether our findings are specific to Egyptian ancestry or generalize to other non-European groups. Third, mechanistic studies examining the role of SIGLEC-1 in renal macrophage infiltration and inflammation could provide insights into LN pathogenesis. While our cohort of 80 SLE patients (47 with LN) is comparable to or larger than many published LN biomarker studies, we acknowledge that the sample size is modest. A post-hoc power calculation for the primary comparison (sSIGLEC-1 levels between LN and non-LN patients) indicates that our sample size provides 80% power to detect a moderate effect size (Cohen’s d = 0.65) at α = 0.05, suggesting that the study was adequately powered for its primary endpoint. However, for subgroup analyses (e.g., comparisons across five WHO classes or five CKD stages), the sample size in individual subgroups was small (e.g., Class III, *n* = 3; Class V, *n* = 4), which may have limited our ability to detect small differences. These subgroup analyses should be considered exploratory, and the negative findings (no association with WHO classes or CKD stages) require validation in larger, multi-center cohorts. We have added this as a limitation to ensure transparent interpretation. Unfortunately, detailed histopathological lesions (e.g., cellular crescents, endocapillary proliferation, fibrinoid necrosis) were not systematically recorded in a quantifiable manner during the original histopathological assessment, as the primary focus of the renal biopsy analysis. So, future studies should specifically examine the correlation between sSIGLEC-1 and these histopathological lesions rather than relying solely on broad WHO classes.

## Conclusion

sSIGLEC-1 is a promising rule-out screening tool for LN in Egyptian SLE patients but is neither a diagnostic test nor a marker of histological severity, proteinuria or chronic renal impairment. Its clinical utility is ancestry-specific and requires careful interpretation within the context of established clinical and histopathological assessments. Future prospective studies in diverse non-European populations are needed to validate these findings.

## Data Availability

All data generated or analyzed during this study are included in this published article.

## References

[1] Koenig, K. F. et al. Serum cytokine profile in patients with active lupus nephritis. *Cytokine* **60**, 410–416 (2012). 22846145 10.1016/j.cyto.2012.07.004

[2] Yap, D. Y. & Lai, K. N. Cytokines and their roles in the pathogenesis of systemic lupus erythematosus: From basics to recent advances. *J. Biomed. Biotechnol.* **2010**, 365083 (2010). 20467470 10.1155/2010/365083 PMC2866250

[3] Hamano, K., Nitta, A., Ohtake, T. & Kobayashi, S. Associations of renal vascular resistance with albuminuria and other microangiopathy in type 2 diabetic patients. *Diabetes Care*. **31**, 1853–1857 (2008). 18566339 10.2337/dc08-0168 PMC2518358

[4] Duan, S. & Paulson, J. C. Siglecs as immune cell checkpoints in disease. *Annu. Rev. Immunol.* **38**, 365–395 (2020). 31986070 10.1146/annurev-immunol-102419-035900

[5] Murugesan, G., Weigle, B. & Crocker, P. R. Siglec and anti-Siglec therapies. *Curr. Opin. Chem. Biol.* **62**, 34–42 (2021). 33607404 10.1016/j.cbpa.2021.01.001

[6] Läubli, H. & Varki, A. Sialic acid–binding immunoglobulin-like lectins (Siglecs) detect self-associated molecular patterns to regulate immune responses. *Cell. Mol. Life Sci.* **77**, 593–605 (2020). 31485715 10.1007/s00018-019-03288-x PMC7942692

[7] Blixt, O. et al. Printed covalent glycan array for ligand profiling of diverse glycan binding proteins. *Proc. Natl. Acad. Sci. USA*. **101**, 17033–17038 (2004). 15563589 10.1073/pnas.0407902101 PMC534418

[8] Heimburg-Molinaro, J., Song, X., Smith, D. F. & Cummings, R. D. Preparation and analysis of glycan microarrays. *Curr. Protoc. Protein Sci.* **64**, 12101–121029 (2011). 10.1002/0471140864.ps1210s64 PMC3097418 21488041

[9] Xiong, Y. S. et al. Increased expression of Siglec-1 on peripheral blood monocytes and its role in mononuclear cell reactivity to autoantigen in rheumatoid arthritis. *Rheumatology* **53**, 250–259 (2014). 24196391 10.1093/rheumatology/ket342

[10] York, M. R. et al. A macrophage marker, Siglec-1, is increased on circulating monocytes in patients with systemic sclerosis and induced by type I interferons and toll-like receptor agonists. *Arthritis Rheum.* **56**, 1010–1020 (2007). 17328080 10.1002/art.22382

[11] Bao, G. et al. Increased Siglec-1 expression in monocytes of patients with primary biliary cirrhosis. *Immunol. Investig*. **39**, 645–660 (2010). 20653431 10.3109/08820139.2010.485625

[12] Lisney, A. R. et al. High maternal expression of SIGLEC1 on monocytes as a surrogate marker of a type I interferon signature is a risk factor for the development of autoimmune congenital heart block. *Ann. Rheum. Dis.* **76**, 1476–1480 (2017). 28501799 10.1136/annrheumdis-2016-210927

[13] Oliveira, J. J. et al. The plasma biomarker soluble SIGLEC-1 is associated with the type I interferon transcriptional signature, ethnic background and renal disease in systemic lupus erythematosus. *Arthritis Res. Ther.* **20**, 152 (2018). 30053827 10.1186/s13075-018-1649-1 PMC6062988

[14] Nasser, M., Wadie, M., Farid, A. & El Amir, A. The contribution of serum sialic acid binding immunoglobulin like Lectin 1 (sSIGLEC-1) as an IFN I signature biomarker in the progression of atherosclerosis in Egyptian systemic lupus erythematosus (SLE) patients. *Ind. J. Clin. Biochem.* **39**, 291–298 (2024). 10.1007/s12291-023-01155-y PMC10987406 38577132

[15] Aringer, M. et al. 2019 European league against rheumatism/American College of Rheumatology classification criteria for systemic lupus erythematosus. *Arthritis Rheumatol.* **71**, 1400–1412 (2019). 31385462 10.1002/art.40930 PMC6827566

[16] Lit, L. C., Wong, C. K., Tam, L. S., Li, E. K. & Lam, C. W. Raised plasma concentration and ex vivo production of inflammatory chemokines in patients with systemic lupus erythematosus. *Ann. Rheum. Dis.* **65**, 209–215 (2006). 15975968 10.1136/ard.2005.038315 PMC1798029

[17] Von Elm, E. et al. The strengthening the reporting of observational studies in epidemiology (STROBE) statement: Guidelines for reporting observational studies. *Ann. Intern. Med.* **147**, 573–577 (2007). 17938396 10.7326/0003-4819-147-8-200710160-00010

[18] Tofighi, T., Morand, E. F. & Touma, Z. Systemic lupus erythematosus outcome measures for systemic lupus erythematosus clinical trials. *Rheum. Dis. Clin. N Am.* **47**, 415–426 (2021). 10.1016/j.rdc.2021.04.007 34215371

[19] Petri, M. et al. Systemic lupus international collaborating clinics renal activity/response exercise (development of a renal activity score and renal response index). *Arthritis Rheum.* **58**, 1784–1788 (2008). 18512819 10.1002/art.23456

[20] Imai, E. et al. Chronic kidney disease Japan cohort (CKD-JAC) Study: Design and methods. *Hypertens. Res.* **31**, 1101–1107 (2008). 18716357 10.1291/hypres.31.1101

[21] Matsuo, S. et al. Revised equations for estimated GFR From serum creatinine in Japan. *Am. J. Kidney Dis.* **53**, 982–992 (2009). 19339088 10.1053/j.ajkd.2008.12.034

[22] National Kidney Foundation. K/DOQI clinical practice guidelines for chronic kidney disease: evaluation, classification, and stratification. *Am. J. Kidney Dis.* **39**, S1–S266 (2002). 11904577

[23] Weening, J. J. et al. The classification of glomerulonephritis in systemic lupus erythematosus revisited. *J. Am. Soc. Nephrol.* **15**, 241–250 (2004). 14747370 10.1097/01.asn.0000108969.21691.5d

[24] Hanley, J. A. & McNeil, B. J. The meaning and use of the area under a receiver operating characteristic (ROC) curve. *Radiology* **143**, 29–36 (1982). 7063747 10.1148/radiology.143.1.7063747

[25] Youden, W. J. Index for rating diagnostic tests. *Cancer* **3**, 32–35 (1950). 15405679 10.1002/1097-0142(1950)3:1<32::aid-cncr2820030106>3.0.co;2-3

[26] Favilli, F. et al. IL-18 Activity in systemic lupus erythematosus. Contemporary challenges in autoimmunity. *Ann. N Y Acad. Sci.* **1173**, 301–309 (2009). 19758166 10.1111/j.1749-6632.2009.04742.x

[27] Tucci, M. et al. Glomerular accumulation of plasmacytoid dendritic cells in active lupus nephritis: Role of interleukin-18. *Arthritis Rheum.* **58**, 251–262 (2008). 18163476 10.1002/art.23186

[28] Strauß, R. et al. Type I interferon as a biomarker in autoimmunity and viral infection: A leukocyte subset-specific analysis unveils hidden diagnostic options. *J. Mol. Med.* **95**, 753–765 (2017). 28357476 10.1007/s00109-017-1515-7

[29] Morimoto, A. et al. Association of endogenous anti-interferon-α autoantibodies with decreased interferon-pathway and disease activity in patients with systemic lupus erythematosus. *Arthritis Rheum.* **63**, 2407–2415 (2011). 21506093 10.1002/art.30399 PMC4028124

[30] Flower, C., Hennis, A., Hambleton, I. R. & Nicholson, G. Lupus nephritis in an Afro-Caribbean population: Renal indices and clinical outcomes. *Lupus* **15**, 689–694 (2006). 17120598 10.1177/0961203306072415

[31] Al Attia, H. M. Lupus Nephritis among Arabs- differences with other races; emphasis on clinicopathological and serological perspectives. *Saudi J. Kidney Dis. Transpl.* **11**, 370–380 (2000). 18209329

[32] Mohsen, M. A., Karim, A., Abbas, S. A., Amin, M. & T. M. & Serum interleukin-18 levels in patients with systemic lupus erythematosus: Relation with disease activity and lupus nephritis. *Egypt. Rheumatol.* **35**, 45–51 (2013).

[33] Kosałka-Wegiel, J. et al. Comparison of clinical and laboratory characteristics in lupus nephritis vs. non-lupus nephritis patients—a comprehensive retrospective analysis based on 921 patients. *J. Clin. Med.* **13**, 4486 (2024). 39124752 10.3390/jcm13154486 PMC11313634

[34] McDonald, S. et al. Predictors of treatment response in a lupus nephritis population: Lessons from the Aspreva Lupus Management Study (ALMS) trial. *Lupus Sci. Med.* **9**, e000584 (2022). 35640982 10.1136/lupus-2021-000584 PMC9157342

[35] Biesen, R. et al. Sialic acid-binding Ig-like lectin 1 expression in inflammatory and resident monocytes is a potential biomarker for monitoring disease activity and success of therapy in systemic lupus erythematosus. *Arthritis Rheum.* **58**, 1136–1145 (2008). 18383365 10.1002/art.23404

[36] Böni, L. et al. *Ann. Rheum. Dis.* **83**, 969–970 (2024).

[37] Hartnell, A. et al. Characterization of human sialoadhesin, a sialic acid binding receptor expressed by resident and inflammatory macrophage populations. *Blood* **97**, 288–296 (2001). 11133773 10.1182/blood.v97.1.288

[38] Faezi, S. T. et al. Clinical picture of lupus nephritis in patients with systemic lupus erythematosus (SLE): Results of a large survey. *Rheumatol. Res.* **2**, 51–59 (2017).

